# Determination of size, sex and maturity stage of free swimming catsharks using laser photogrammetry

**DOI:** 10.1007/s00227-017-3241-7

**Published:** 2017-10-23

**Authors:** Toby D. Rogers, Giulia Cambiè, Michel J. Kaiser

**Affiliations:** 1 School of Ocean Sciences, Bangor University, Anglesey, LL59 5AB UK; 2 Centre for Environment Fisheries and Aquaculture Science (CEFAS), Lowestoft, Suffolk NR33 0HT UK

## Abstract

The lack of detailed life history (LH) information (e.g. age, growth, size at maturity, sex composition etc.) for many species of conservation importance limits the implementation of appropriate conservation measures. Typically, LH information is acquired using lethal sampling techniques, which undermines the goal of conservation. This is particularly problematic for many shark species that have low fecundity and slow growth rates. Here we tested the use of non-invasive laser photogrammetry to measure body morphometry in vivo. We used random forest classification models to identify allometric relationships (ratios between body measurements) that discriminated between the sex and stage of sexual maturity of *Scyliorhinus canicula*. We coupled the use of allometric ratios (determined from cadavers) with parallel laser photogrammetry, in order to collect total length (TL) and finer scale morphometrics from 37 free-swimming individuals. TL measurements proved to be accurate (SE = 5.2%) and precise (CV = 1.8%), and did not differ significantly from the known TL of the respective animal (*t*36 = 0.7, *P* = 0.5). Conditional Inference tree model predictions of free-swimming sharks correctly predicted 100% of mature males and 79% of immature males. Our results suggest that when used together, allometric ratios and parallel laser photogrammetry have the potential to be a promising alternative to collect essential life history information from free swimming animals and avoids the need for destructive sampling.

## Introduction

Over the last century, many of the world’s elasmobranch populations have faced declines, through a combination of overexploitation by commercial fishers (targeted and by-catch) and a lack of effective fisheries management (Dulvy et al. 2003; Baum and Myers 2004; Heupel and Simpfendorfer 2010; Worm et al. 2013). Many elasmobranchs are particularly vulnerable to sustained levels of exploitation and have a low recovery potential, because they have slow growth rates, low fecundity and late onset maturity (Musick et al. 2000; Simpfendorfer et al. 2000). Life history parameters (e.g. size at maturity, growth function, etc.), size distribution, are all crucial knowledge needs in order to improve the assessment of a species’ population status and for the implementation of effective management strategies (Camhi 1998; Baum et al. 2003; Labrada-Martagón et al. 2014). Traditional methods for the collection of quantitative life history data are typically destructive and have the potential to undermine the conservation efforts for vulnerable species (Deakos 2010; Varghese et al. 2016).

The use of non-lethal sampling techniques is desirable to gather quantitative data on reproductive biology and sex of elasmobranchs (Awruch et al. 2008; Hoffmayer et al. 2010; Whittamore et al. 2010). However, even the use of techniques such as steroid hormone analysis and ultrasonography require individuals to be caught and manipulated to conduct the assessment. The capture process inevitably increases physiological stress levels which may result in post-release mortality of more sensitive species (Revill et al. 2005; Skomal 2007; Molina and Cooke 2012; Gallagher et al. 2014).

Ontogenetic changes in allometry can be used to identify specific developmental stages and potentially sex, if sexual dimorphism of body allometry is present (Abouheif and Fairbairn 1997). The use of ratio measurements has previously been employed to distinguish between morphologically similar species as well as quantify stages of sexual maturity for teleosts and crustaceans (Fernández-Vergaz et al. 2000; Nikolioudakis et al. 2010; Baur and Leuenberger 2011; Ribeiro et al. 2013). For most commercial fishes and crustaceans multiple samples for morphometric analysis are easy to collect through destructive sampling techniques without impacting further on the population (i.e. using catches from commercial vessels or scientific surveys as opportunistic data sources). However, for large elasmobranchs the use of targeted and destructive sampling is not usually possible (except perhaps for landed bycatch). Thus, the development of a methodology for data collection through non-destructive measurement of morphometric variation in individual animals using techniques that can be deployed in situ would be highly desirable.

Recent refinements of photogrammetric techniques have enabled precise measurements of body features to be captured with relative ease with no physiological cost to the animal (Deakos 2010). As opposed to more typical in vivo visual estimations of size and sex by trained observers, photogrammetry offers the potential for greater degrees of accuracy, precision and repeatability (Norman and Stevens 2007). Although some variants of the technique require arduous calibrations (Klimley and Brown 1983; Harvey et al. 2002a, b) it is adaptable to research needs and can be utilised in remote environments. Parallel Laser Photogrammetry is a simple and relatively cheap modification of a single camera arrangement, with the addition of a parallel laser array. The projection of a length laser bar or pairs of points (a fixed distance apart) onto the flank of an animal can be analysed with the use of image analysis software, such that accurate length and morphometric data can be collected from still images (Webster et al. 2010; Rohner et al. 2011). To date, a handful of studies have successfully utilised parallel laser photogrammetry to gather simple length measurements of the whale shark (*Rhincodon typus*) (Rohner et al. 2011, 2015), and the great white shark (*Carcharodon carcharias*) (Leurs et al. 2015). The possibility of using laser photogrammetry to effectively categorise sex and maturity stage would be provide valuable insights into an elasmobranch’s population structure. Furthermore the technique is potentially applicable to any large-bodied fauna for which sex and maturity can be inferred from external body metrics.

The aims of the present study were to combine the use of ontogenetic allometry ratios with parallel laser photogrammetry under controlled conditions to; (i) identify morphometric ratios that accurately predicted both the maturity stages (i.e. mature and immature) and sexes of a model species of shark, (ii) quantify the error associated with parallel laser photogrammetry length estimates relative to real measures from the same individual, and, (iii) integrate the two techniques to determine the viability of non-invasively predicting the sex and maturity stage of free-swimming individuals.

## Methods

We used the small-spotted catshark (*Scyliorhinus canicula*) as a model elasmobranch species. This oviparous catshark is the most abundant shark in EU waters (Ellis and Shackley 1997). We obtained 101 dead *S. canicula* (46 males and 55 females) collected from the bycatch of a commercial scallop dredge-fishing vessel (June 2015). Thirty seven live specimens were collected for tank-based photogrammetry data collection as part of the annual fisheries survey in Red Wharf Bay and Conwy Bay conducted by the RV Prince Madog (October 2016) (Table 1). We were unable to obtain female *S. canicula* samples for tank analysis. The sexual composition of the catch could be attributed to factors such as season or differential behaviour strategies displayed between the sexes, where *S. canicula* are known to segregate by sex (Ford 1921, Sims et al. 2001).

**Table 1:** Total length (TL) (mm) ranges of dead and alive *Scyliorhinus canicula* specimens relative to maturity classification and the respective sample size (*n*) at each maturity stage

	Male	Female
Dead		
Immature	219.0–486.0 (16)	262.0–526.5 (26)
Mature	484.5–664.5 (30)	470.5–628.0 (29)
Alive		
Immature	213.0–468.0 (16)	–
Mature	501.0–650.0 (21)	–

**Fig. 1:**
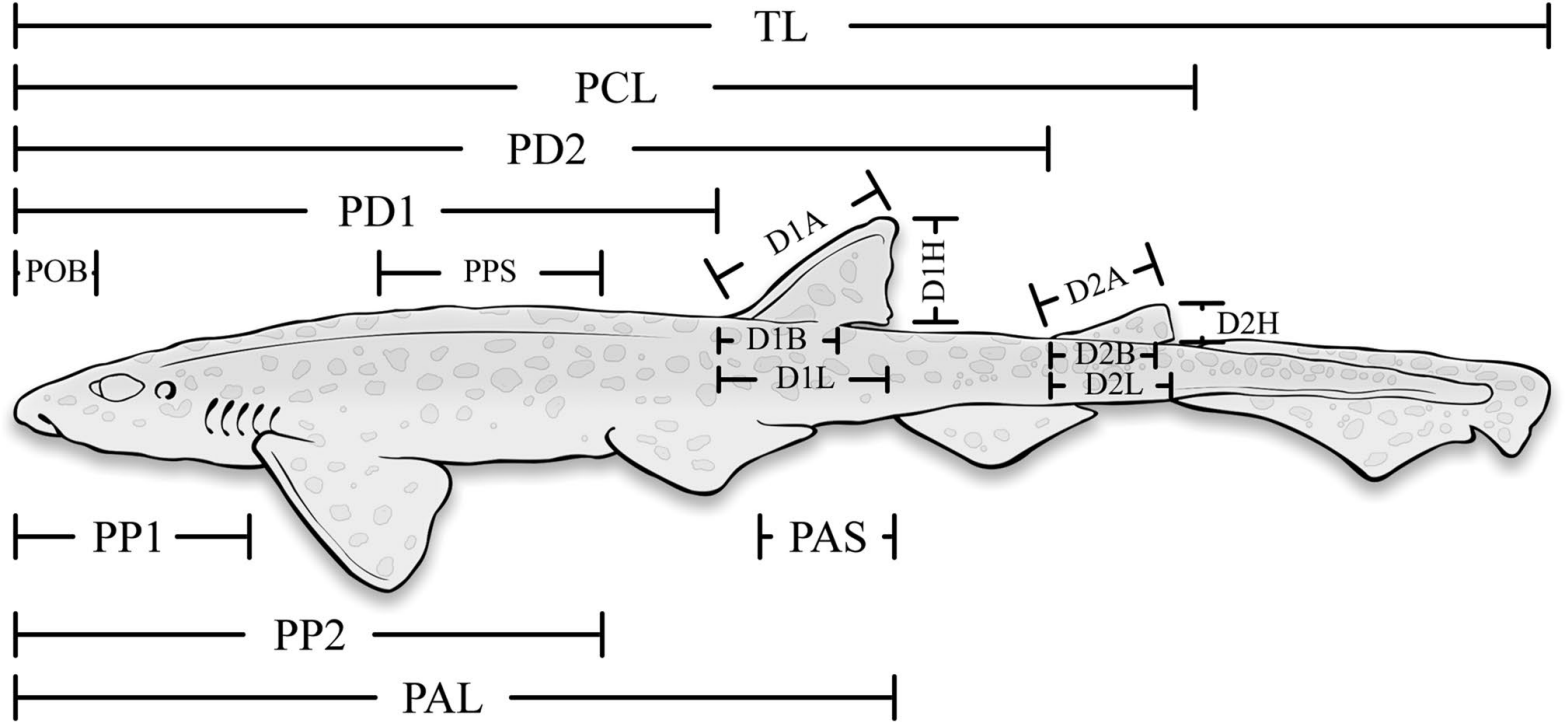
Illustration of the model species (*Scyliorhinus canicula*) with the morphometric measurements according to Compagno (2001) overlain. Only the morphometrics that appear in the final analysis have been detailed here. Acronyms correspond to those outlined in Table 2

In preparation for measurements, frozen *S. canicula* were thawed, sexed and weighed to the nearest 0.1 g. We collected morphometric measurements according to metrics described by Compagno (2001) (Fig. 1; Table 2) and following the protocols in Mollet et al. (1996) and Bass (1973). Morphometric dimensions were selected a priori to maximise the possibility of measuring these from still images captured from digital video. Each specimen was placed in a natural position (Compagno 2001) on a fish board to collect morphometric measurements (± 1 mm). Measurements were collected from both sides of each specimen and averaged to reduce measurement errors.

**Table 2:** The external morphometric measurements collected from specimens of *Scyliorhinus canicula* following the protocol in Compagno (2001)

Acronym	Morphometric	Acronym	Morphometric
TL	Total Length	PAS	Pelvic-Fin Anal-Fin Space
PCL	Precaudal-Fin Length	D1L	First Dorsal-Fin Length
PD1	Pre-First Dorsal-Fin Length	D1A	First Dorsal-Fin Anterior Margin
PD2	Pre-Second Dorsal-Fin Length	D1B	First Dorsal-Fin Base Length
POB	Preorbital Length	D1H	First Dorsal-Fin Height
PP1	Prepectoral-Fin Length	D2L	Second Dorsal-Fin Length
PP2	Prepelvic-Fin Length	D2A	Second Dorsal-Fin Anterior Margin
PAL	Preanal-Fin Length	D2B	Second Dorsal-Fin Base Length
PPS	Pectoral-Fin Pelvic-Fin Space	D2H	Second Dorsal-Fin Height

The acronyms defined below are used throughout the remainder of the paper

The maturity stage of each specimen was determined from macroscopic examination of the gonads. The maturity criteria defined by Capapé et al. (2014) for *S. canicula* together with the direct observations made in this study enabled discrete descriptions to be formulated for each maturity classification (Table 3).

**Table 3:** Description of the anatomical and morphological characteristics of each maturity stage for *Scyliorhinus canicula* adapted from Capapé et al. (2014) and observations made in this study

	Male	Female
Immature	Undeveloped and thread like genital ducts, short and flexible claspers retained within the pelvic fins	Ovaries undeveloped with thin oviducts and small unremarkable oviducal glands
Mature	Well-developed testes, genital duct and sperm present in the seminal vesicles (depress posterior opening (cloaca) with thumb to test). Claspers well developed calcified and protrude slightly outside the pelvic fins, claspers occasionally red in colour	Fully developed genital ducts and ovary which contains yolky oocytes. Large rounded oviducal glands. Egg cases present in oviducts of running adults

The Parallel Laser Photogrammetry (hereafter referred to as PLP) array consisted of a pair of underwater red lasers (Tritech UK, class 3R < 4 mW 650 nm) separated by a fixed distance of 100 mm and a GoPro Hero 3+ silver edition video camera, programmed to film high-resolution video (1080p) at a frame rate of 30 fps. The medium lens angle was selected which enabled a field of view (FOV) of 127°, and in-turn reduced the fish-eye distortion effect associated with wide-angle lenses. The array was positioned against the wall at one end of the observation tank (3 m in length, 0.75 m wide and 0.6 m deep), with seawater at ambient temperature and was filtered over a 0.1 µm mesh to reduce the amount of particulate matter in the water column.

In order to identify the accuracy and precision of image measurements, a cylindrical plastic pipe of known length (500 mm) was placed in the tank at set distances away from the camera (500, 750, 1000, 1250, 1500, 1750 and 2000 mm). The mean percent error and the coefficient of variation (CV) of repeated measurements (*n* = 3) were used as measures of accuracy and precision of the length estimates respectively. Multiple studies have highlighted the parallax errors introduced when an object deviates from perpendicular to the FOV of the camera (e.g. Deakos 2010; Webster et al. 2010; Rohner et al. 2011). We collected length measurements of the same pipe at incremental angles of 10° from 50° to 90°, in order to further evaluate the error introduced for measurements that deviated from the perpendicular FOV of the camera. In addition, periodic calibrations were performed to ensure that the lasers were correctly aligned.

Live *S. canicula* were stored in holding tanks with seawater and held at ambient temperature. Individuals were placed one at a time into the observation tank where video footage was collected. Lasers were directed toward a stable position on the body of individual free-swimming *S. canicula* (between the fifth gill slit and first dorsal fin) (Leurs et al. 2015). Once all video data were collected for an individual, it was removed and measured by hand on a damp fish board. To minimise stress to each fish, an ethical and safe technique to restrain each individual was utilised (Henningsen 1994). Specimens were brought to the surface and inverted dorso-ventrally whilst submerged until the body relaxed (time elapsed ~ 60–90 s), whereupon each individual was removed from the tank, measured to the nearest mm. Maturity for live males was determined by evaluating the level of calcification (rigidity) and length of the claspers by hand (Awruch et al. 2014). Individuals were returned to holding tanks and typically regained mobility after 20–30 s. Handling procedures followed ethical committee guidelines of Bangor University.

A minimum of three still images were collected from the video footage for each individual, provided images were perpendicular to the FOV, not pixelated, and had sufficient lighting. ImageJ was used to measure morphometric data from each individual photograph. The laser points projected onto the target allowed us to calibrate each image and collect measurements in mm.

### Data analysis

Morphometric ratios were calculated by dividing the length of one body part by another, according to morphometric characteristics listed in Table 2. To assess the presence of different morphometric ratios between mature and immature fish by sex, and between males and females, a random forest classification model (R package ‘randomForest’ (Liaw and Wiener 2002)) was used. Random forest (RF) is a non-parametric method based on decision-tree modelling approach (Breiman 2001), which requires fewer assumptions than traditional parametric methods (e.g. linear discriminant analysis; Strobl et al. 2009). In particular RF allows correlated predictor variables (in our case the morphometric ratios) to be used without transformation or exclusion to obtain unbiased predictions and estimates of variable importance (Strobl et al. 2009). Random forest analysis has been shown to provide discrimination based on otolith microchemistry and scales stable isotopes when the assumptions of the traditional parametric methods cannot be met (Mercier et al. 2011; Cambiè et al. 2016). The random forest model produces many classification trees from which are derived an ensemble of classifications to predict the dependent variable (in our case ‘maturity stage of males and females (mature vs immature)’ and ‘sex’ of *S. canicula*) as a result of average assignment across trees (Strobl et al. 2007, 2009). By default, the random forest model partitions the data into ‘training’ (generally 70% of data) and ‘test’ samples, randomly selected from the data set. The training samples are used to build the model and the test set is used to validate its performance.

The random forest classification model was then used to test for differences in morphometric ratios among males and females and among mature and immature fish for each sex and whether it was possible to correctly assign the fish to its maturity stage and sex based on these morphometric ratios. In addition, this statistical technique allowed the importance of each predictor variable (each morphometric ratio) in the classification process to be evaluated and ultimately to identify specific morphometric ratios for each sex and maturity stage. Three RF models were run (males vs females, immature males vs. mature males and immature females vs mature females). Each model was run twice. The first run included all combinations of morphometric ratios, which allowed us to identify the most important ratios for classifying fish by sex and maturity stage. To this end, the conditional variable importance was calculated to investigate the relative contribution of each predictor (morphometric ratios) to the classification performance and thus to identify the top ten most important morphometric ratios for each model. To evaluate the conditional variable importance, we measured the mean decrease accuracy (MDA) of the forest when the values of each predictor were randomly excluded (or permuted). The greater the decrease in the accuracy of the random forest resulting from the exclusion (or permutation) of a single variable, the more important that variable is for classification of the data. Each model was then run including only the top ten morphometric ratios identified, and thus the ratios that were most important in terms of discrimination capacity between sex and maturity. This also allowed us to specify which morphometric ratios to focus on when collecting measurements from still images of individuals a priori, saving time when processing images.

Finally, to aid interpretation of the results of the random forest analysis, a conditional inference tree was used. This analysis was focused only on the RF models characterised by a good prediction performance and enabled to identify the range of the morphometric ratios values associated with each sex and/or maturity stage of male and female. The ‘ctree’ function for conditional inference trees in the ‘party’ R package (Hothorn et al. 2006) was used. Live fish (males) were then used to test if the trade-off values of the morphometric ratios identified in the conditional inference tree enabled a correct classification of the maturity stage.

Since measurements of TL are affected by the body flexion of sharks during normal swimming, it was important to identify the most stable and reliable morphometric predictors of TL (Rohner et al. 2011). We analysed three different stable morphometrics from the dorsal fin; D1L, D1A and D1H from dead specimens with linear regression, in order to identify the strongest predictor of TL. Using the strongest TL regression relationship equation, we plotted the estimated TL from the still images (with 95% CI) against the known TL of the individual. A paired *T* test was employed to identify whether significant differences between estimated and known measurements were evident. This allowed us to understand whether our TL estimates of free-swimming sharks were in agreement with the known TL of each individual and to demonstrate the true accuracy of PLP when measuring animals, something that to the best of our knowledge has not been quantified to date.

## Results

The random forest classification model built on the training samples (total number of ratios analysed for each model = 117) showed good discrimination capacity, being able to correctly classify the sex (80%) and the maturity stage for males (80%) and for females (78%). The conditional variable importance (mean decrease in accuracy) identified the ratios that best predicted sex and maturity, in order of most to least important. From this the top 10 ratios were selected in an attempt to focus on only the most important ratios. Upon removal of the bulk of the least important ratios and rerunning the models, the discrimination capacity for sex remained similar to the overall model (79%) while only slight improvements were observed for both male (86%) and female (83%) maturity (Table 4).

**Table 4:** The top 10 ratios highlighted by the mean decrease in accuracy (MDA), for the random forest classification models of sex and maturity stage, for male and female *Scyliorhinus canicula*

Sex	MDA	Male maturity	MDA	Female maturity	MDA
PPS.PAS	7.45	PD1.PD2	6.12	TL.PPS	6.86
PP2.PAS	6.48	TL.PD1	5.95	PCL.PPS	6.35
PCL.PAS	6.30	PCL.PD1	4.91	TL.PP2	5.38
PD2.PAS	5.61	PP1.D2A	4.83	PPS.D2L	5.30
PP1.D2H	5.28	PD1.D2A	4.19	TL.PD2	4.97
PAL.PAS	5.18	PD1.D2B	3.84	PP2.PPS	4.52
TL.PAS	5.07	PP1.D1L	3.78	TL.PD1	4.38
PP1.D1L	4.98	PP1.D2L	3.62	PD2.PPS	4.38
PPS.POB	4.98	PD1.D1A	3.42	PP2.D2L	3.46
POB.D2H	4.43	PPS.D2A	3.30	PCL.PD1	3.19

Consistency in the classification accuracy between the models built on training and testing data was found for sex classification and maturity stage of male fish, which confirmed a good prediction performance of the model. (Table 5). However for females, the RF for the test data, showed an error rate higher than that obtained from the model built on training data. This discrepancy was observed in both the model discerning males from females (in the test data, the  % of females classified as males was higher than in the training data set, Table 5) and the model that classified mature and immature female fish.

**Table 5:** Random forest classification models detailing the observed versus predicted sex (*M* male, *F* female) and maturity stage relative to sex (*Imm* immature, *Mat* mature) for *Scyliorhinus canicula* for training and testing (cross-validation) data sets

	Predicted				Error (%)	
	Train	Test	Train	Test	Train	Test
Observed						
Sex	*M*	*M*	*F*	*F*		
*M*	**25**	**8**	10	3	28.6	27.3
*F*	6	4	**36**	**9**	14.3	30.8
Male	*Imm*	*Imm*	*Mat*	*Mat*		
*Imm*	**9**	**4**	3	0	25.0	0.00
*Mat*	2	1	**21**	**6**	8.70	14.3
Female	*Imm*	*Imm*	*Mat*	*Mat*		
*Imm*	**15**	**5**	4	2	21.1	28.6
*Mat*	3	2	**19**	**5**	13.6	28.6

Correct model prediction values are denoted in bold, with the respective percentage error of each model

**Fig. 2:**
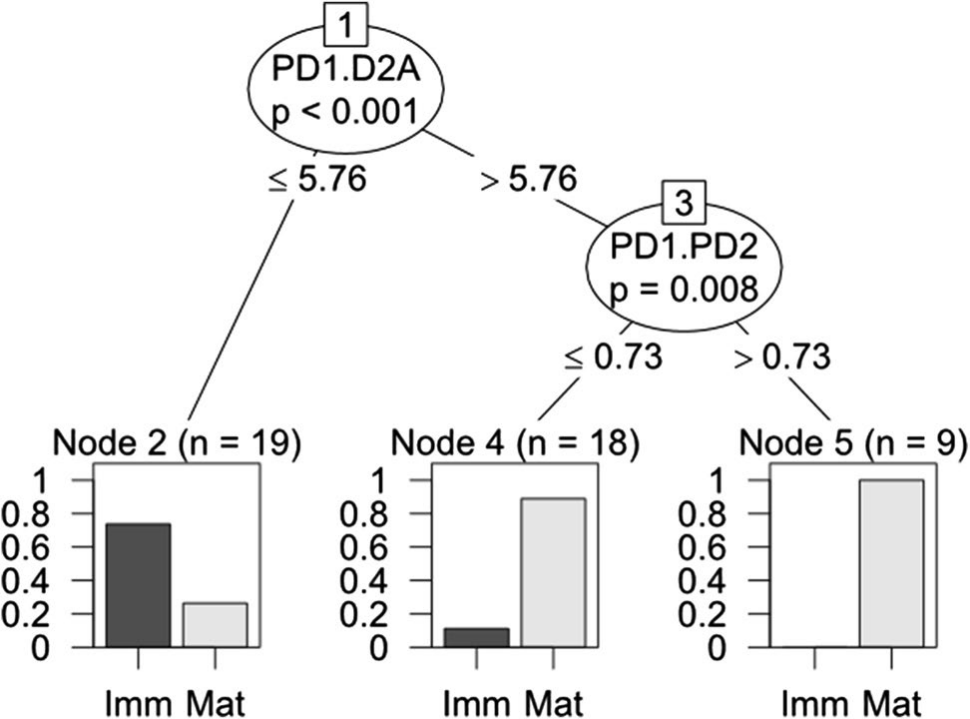
Conditional inference tree which highlights the discriminatory morphometric ratios used to predict the maturity stage (*Imm* immature, *Mat* mature) of male *S. canicula* (combined train *n* = 35 and test *n* = 11 data) Inner nodes (ovals) specify significantly different ratios used for splitting the tree towards the terminal nodes, which are expressed as bars relative to the proportions immature/mature sharks. Inner node acronyms; PD1.D2A equate to—the ratio between the Pre-First Dorsal Fin Length and the Second Dorsal-Fin Anterior Margin, PD1.PD2 to—the ratio between the Pre-First Dorsal Fin Length and the Pre-Second Dorsal-Fin Length

The conditional inference tree was focused on the model that discerned between mature and immature fish male, as it was characterised by the best prediction accuracy. The most important primary split in the conditional inference tree was the PD1.D2A value; most of the mature fish (87%, *n* = 26) were allocated on the branch corresponding to PD1.D2A ≥ 5.76 (Fig. 2). In particular, 100% (*n* = 9) of fish with PD1.D2A ≥ 5.76 and PD1.PD2 > 0.73 were mature. 94% of the immature male fish were characterised by PD1.D2A < 5.76. The ratio between the pre-first and the second dorsal fin length thus appeared to be the most important value for a good prediction of the maturity stage for male fish.

To identify the best predictor of TL for *S. canicula*, we analysed three morphometrics with linear regression. Although the TL-D1A (male: TL = 0.094D1A + 5.82, *R* ^2^ = 0.90, female: TL = 8.73D1A + 35.19, *R* ^2^ = 0.87) and TL-D1H (male: TL = 0.057D1H–0.34, *R* ^2^ = 0.87, female: TL = 13.32D1H + 116.27, *R* ^2^ = 0.85), relationships were strong, the TL–D1L relationship for both sexes proved to be the strongest proxy for TL. This regression relationship was selected to estimate the TL of sharks from still images.

**Fig. 3:**
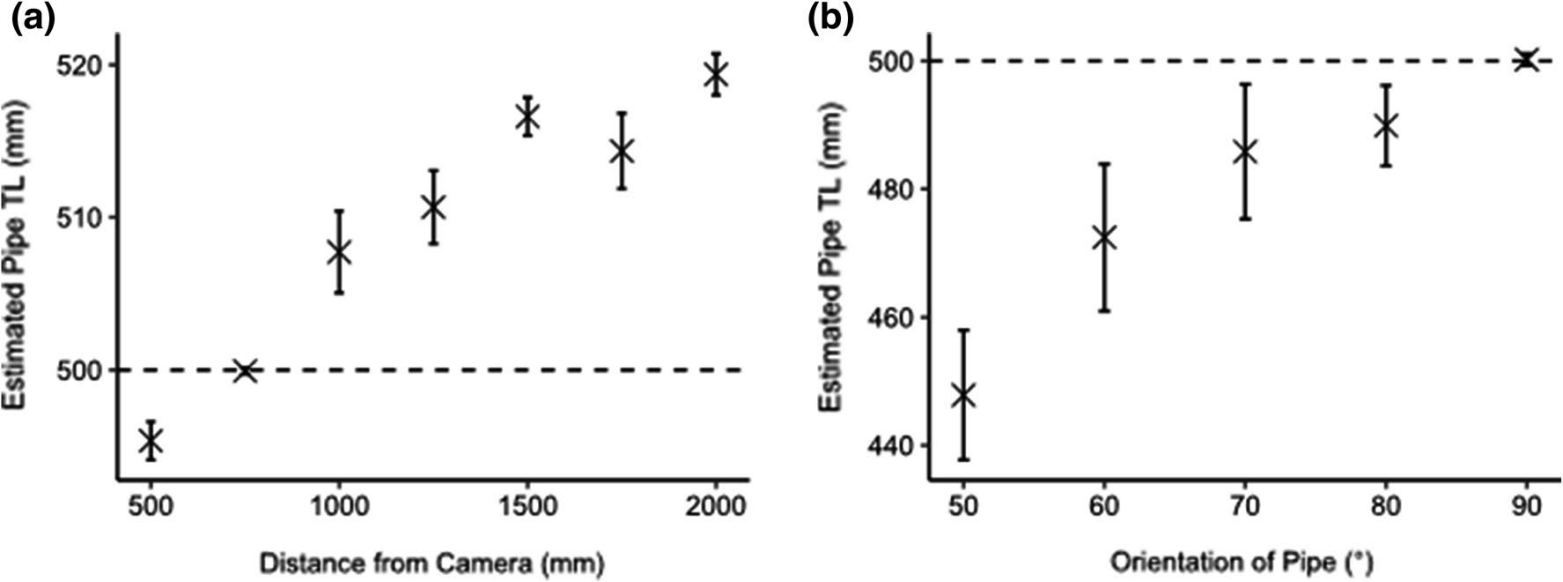
The mean estimated total length (TL) (mm ± 95% CI) of a 500 mm pipe measured by positioning the Parallel Laser Photogrammetry (PLP) array at seven different distances, from 500 to 2000 mm (distance increment = 250 mm) (**a**), and the mean estimated total length (mm ± 95% CI) of the same pipe measured by positioning the PLP array at incremental angles of 10° away from the perpendicular field of view of the camera (**b**). The dashed lines reference the known length of the pipe

The estimated TL of the plastic pipe was most accurately (SE = 0.1%) and precisely (CV = 0.04%) evaluated at a distance of 750 mm from the camera (Fig. 3a). A general decrease in TL estimate accuracy was observed with increased distance from the camera; therefore we only collected TL and morphometric measurements of free-swimming individuals at the most accurate distance from the camera (i.e. 750 mm). The error associated with measurements collected at a distance of 500 mm can be attributed to how close the pipe was to the camera array. At this distance the ends of the pipe were recorded at the edge of the image frame, where optical distortion is greatest and thus increased measurement error at this distance. Our results also demonstrated the importance of collecting TL measurements at a perpendicular angle to the FOV of the camera array, where deviations from 90° yielded underestimates of TL and an increase in measurement variability (Fig. 3b).

**Fig. 4:**
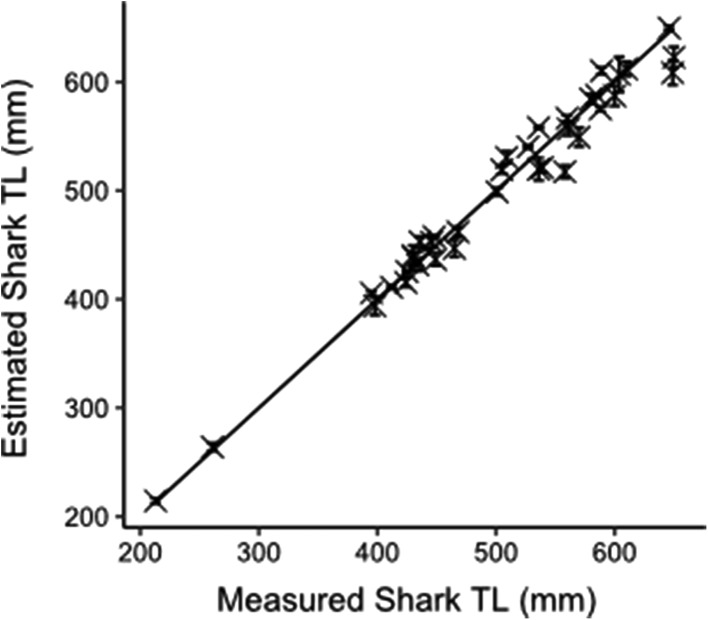
Estimated TL (from the TL–D1L relationship, TL = 10.55D1L + 18.53) vs measured TL of the 37 free-swimming sharks (solid line indicates 1:1 measured TL relationship). Estimated TLs (mm ± 95% CI) were averaged across three replicate images of each individual obtained with the PLP array at a distance of 750 mm

Estimates of TL were made for 37 live male *S. canicula* using PLP (Fig. 4). D1L was measured from three still images and an average calculated for each individual free-swimming shark to estimate TL. A paired T-test demonstrated that there was no significant difference observed between the mean TL of the estimated and measured sharks (*t*36 = 0.7, *P* = 0.5). In addition, the accuracy (SE = 5.2%) and precision (CV = 1.8%) of the TL measurements were high, and therefore allowed us to proceed to collect finer scale morphometric measurements.

Estimates of PD1, D2A and PD2 were collected from the still images of the free-swimming sharks, to test the discrimination capacity between mature and immature males from the CI tree analysis. These estimates were collected from 26 males (14 immature, 12 mature), due to the poor quality of some images. The trade-off values of the ratios PD1.D2A and PD1.PD2 obtained from the CI tree model built on the 46 dead male sharks correctly predicted the maturity stage of 88% of the free-swimming fish (Table 6). The best prediction was for mature males (100% fish correctly assigned), while for immature fish the accuracy decreased to 79%, which represents, in any case, a very good prediction score (Table 6).

**Table 6:** Predictions of the maturity stage (immature/mature) for 26 male free-swimming *Scyliorhinus canicula* (of known maturity) obtained by using the tree trade-off values from the CI tree analysis built on the 46 dead males

	Predicted		
	Immature	Mature	% Error
Observed			
Immature	**11**	3	21
Mature	0	**12**	0

Correct model prediction values are denoted in bold, with the respective percentage error of each model

## Discussion

Current techniques that attempt to evaluate biological information non-invasively are either unable to provide the same levels of data yielded through destructive sampling, or only able to yield information about single biological metrics (Heupel and Simpfendorfer 2010; Whittamore et al. 2010; Hammerschlag and Sulikowski 2011). Here, we have attempted to bridge this gap by integrating the use of allometric growth ratios with laser-photogrammetry to quantify multiple aspects of shark biology, under controlled conditions.

Sexual size and morphometric dimorphism is a common trait in the animal kingdom, where elasmobranchs and more specifically *S. canicula* are no exception to this (Abouheif and Fairbairn 1997; Ellis and Shackley 1997; Sims 2005; Filiz and Taskavak 2006). While changes in allometry have been used to detect the onset of maturity in other species, this is the first evidence that it is applicable for sharks (Abouheif and Fairbairn 1997; Nikolioudakis et al. 2010; Baur and Leuenberger 2011). Typically, size/shape or morphometry data are highly correlated and previous studies have analysed data using a relatively convoluted principle component analysis or linear discriminate analysis technique, to overcome correlative behaviour (Williams et al. 2015). Our random forest results provide early evidence that allometric ratios have the potential to discriminate between the sex and maturity stage of a small elasmobranch non-invasively. In particular, our model results and predictions performed very well for male maturity. We identified specific trade-off values for a combination of allometric ratios that demonstrated a robust level of discrimination between immature and mature males, as well as a combination of ratios that were typical of only mature males (PD1.D2A > 5.76 and PD1.PD2 > 0.73). We acknowledge however that the issue of the sample size imbalance could also be a factor here. On the other hand, female maturity was less clearly classified by our analysis. Four of the ratios highlighted by the MDA analysis comprised measurements of TL, and we suggest that the larger overlap in TL between maturity stages for females could account for some of the misclassification error here (exclusion of TL measurements from analysis destabilised model results).

Our parallel laser photogrammetry approach proved to be accurate, precise and did not differ significantly from the known TL of each individual. This is the first study to directly compare estimated measurements of TL with the known length of the same individual, for free-swimming marine species. Typically photogrammetry studies use an inanimate object of a known size to quantify the error of photogrammetric measurements and apply a correction factor to account variables such as lens distortion, with the assumption that error is uniform (Deakos 2010; Rohner et al. 2011; Leurs et al. 2015). Here however, we demonstrated that our morphometric proxy for TL, although an accurate proxy, did not yield a uniform over or underestimation of TL, which would bring into question the validity of correction factors for future photogrammetry studies. While we accept that in practice it is difficult to collect measurements from a fixed distance from the camera array (especially for divers), we emphasise the importance of taking camera distance into consideration. Here we demonstrated that over a small range of distances from the camera (1.5 m), estimation error of an inanimate object begins to increase quite considerably, and will therefore introduce uncertainty towards the true measurements of free-swimming animals. Perhaps more importantly, we along with other studies (e.g. Deakos 2010), emphasise the need for measurements to be collected from individuals that present themselves in a perpendicular plane to the FOV of the camera, as measurement accuracy can be vastly compromised if not adhered to. Despite this, the accuracy, precision and repeatability of photogrammetric measurements present a much better alternative to yield TL data, as opposed to relying on trained observer estimates (Harvey et al. 2002a, b; Rohner et al. 2011). Another photogrammetric technique to consider is the use of stereo cameras. Stereo-photogrammetry has the potential to yield increased measurement accuracy when compared to the single camera counterpart, but its uptake has typically been outweighed by the cumbersome nature of calibration, deployment and image analysis, as well as cost (Klimley and Brown 1983; Dawson et al. 1995, Harvey et al. 2002b). Recent technological advances have brought down costs, and likewise dedicated software (e.g. SeaGIS) has allowed for more efficient analysis of images (Boutros et al. 2015; Letessier et al. 2015; Delacy et al. 2017). Yet, the lower learning curve and budget required to operate parallel laser photogrammetry arrays may be more favourable to some researchers needs than stereo-photogrammetry.

Intraspecific variability around the length or age a species reaches before the onset of sexual maturity is in part driven by environmental stressors, and therefore TL at maturity is inherently variable (Stearns and Crandall 1984; Saunders and McFarlane 1993). Here we have demonstrated that allometric growth ratios have the potential to classify the sex and stage of sexual maturity for dead specimens. Thus we coupled allometric growth ratios with our accurate laser photogrammetry technique to predict the maturity stage of free-swimming male sharks. Unlike many laser photogrammetry field studies, we were able to validate our model predictions as we knew the maturity stage of each shark (through examination of the claspers). While our results were limited to predicting the maturity stage of free-swimming male *S. canicula* due to sample availability (where variation in the sex ratio of landings through the year is a common feat (Ford 1921)), the models presented a high degree of prediction accuracy, which suggests the PLP and allometric growth ratio combination could be a viable alternative to destructive sampling. While some authors argue that lethal sampling through fishery dependant or independent sources remains the most accurate method to amass life history data from a species (even threatened species) (Heupel and Simpfendorfer 2010), we stipulate that our approach here has the ability to offer an alternative quantitative tool for researchers (Hammerschlag and Sulikowski 2011). Furthermore, with increasing uptake of Baited Remote Underwater Video Systems (BRUVS) or diver based surveys, for long term monitoring of a diverse range of marine species and environments, we envisage that our technique could be integrated with current camera arrays with relative ease (Cappo et al. 2003; Lorenzo et al. 2011; Walsh et al. 2016). We propose that the life history data our technique is able to evaluate, will be a useful addition to studies that are collecting information about individual site fidelity using PhotoID, abundance estimates, long term growth rates, as well as more broadly community species richness and diversity, and would be particularly suited to collect data from within marine reserves. For the photogrammetry array that we have proposed here, we suggest that it would be most suited to assess small-medium sized elasmobranch species either attracted to the array (e.g. laser-photogrammetry BRUV) or operated by a diver, due to the measurement error introduced beyond 1 m from the array. Beyond this distance and for larger species, a researcher could look at modifying the array where appropriate to include higher quality cameras to collect data from greater distances, although we suggest the quality control procedures carried out here be followed. Alternatively, since the RF model presented here just requires finer scale morphometric measurements, it is entirely feasible to integrate the use of allometric growth ratios into already established photogrammetry studies. Here we demonstrated the use of allometric growth ratios in the context of elasmobranch biology, but PLP techniques are being employed in both the terrestrial and marine realms. Therefore we envisage that with some methodological refinement, our complementary approach has broad and adaptable applications for use with other vulnerable species.

In conclusion, we have demonstrated that photogrammetry has the ability to yield more LH information from an individual than just TL. The evidence from this study provides a promising alternative to yield sex and maturity data from free-swimming sharks, although the data were clearer when determining male sex and maturity. While we accept that our study was conducted under controlled conditions and concerns a relatively small species of shark, we argue that it was important to validate the utility and accuracy of this technique to estimate size, sex and maturity before applying the technique to species with greater conservation concern. For the first time, we also evaluated the PLP measurement accuracy associated with free-swimming sharks. The results here were important, because although not significantly different, measurements collected at the optimal distance did not have a uniform pattern of over/under estimation. This is noteworthy for studies that look to account for measurement error with uniform correction factors. Furthermore, we suggest that our method of using allometric ratios and PLP could be integrated into current diver based or remote monitoring investigations with relative ease and could be applied to a wide range of species that have external morphological differences related to sex and maturity. Further investigations should be carried out with species that have stable populations (i.e. destructive sampling) first in order to validate the wider applicability of the technique before considering vulnerable species. If validated, then the need to sacrifice animals can be removed and models for vulnerable species can be built with a combination of historical morphometric data and incidental bycatch of a study species.
